# Activated Carbon-Incorporated Tragacanth Gum Hydrogel Biocomposite: A Promising Adsorbent for Crystal Violet Dye Removal from Aqueous Solutions

**DOI:** 10.3390/gels9120959

**Published:** 2023-12-07

**Authors:** Badr M. Thamer, Faiz A. Al-aizari, Hany S. Abdo

**Affiliations:** 1 Department of Chemistry, College of Science, King Saud University, P.O. Box 2455, Riyadh 11451, Saudi Arabia; faizalaizari@yahoo.com; 2 Department of Mechanical Engineering, College of Engineering, King Saud University, Riyadh 11421, Saudi Arabia; habdo@ksu.edu.sa

**Keywords:** tragacanth gum, activated carbon from pomegranate peel, hydrogel composite, crystal violet adsorption, Sips model, wastewater treatment

## Abstract

Biomaterials-based adsorbents have emerged as a sustainable and promising solution for water purification, owing to their eco-friendly nature and remarkable adsorption capacities. In this study, a biocomposite hydrogel was prepared by the incorporation of activated carbon derived from pomegranate peels (PPAC) in tragacanth gum (TG). The hydrogel biocomposite (PPAC/TG) showed a porous structure, a negative surface charge at a pH of more than 4.9, and good stability in aqueous media. The adsorption properties of the PPAC/TG hydrogel biocomposite were assessed for the removal of crystal violet dye (CV) from aqueous solutions using a batch adsorption. The equilibrium adsorption data followed the Sips isotherm model, as supported by the calculated R^2^ (>0.99), r-χ^2^ (<64), and standard error values (<16). According to the Sips model, the maximum values of the adsorption capacity of PPAC/TG were 455.61, 470.86, and 477.37 mg/g at temperatures of 25, 30, and 35 °C, respectively. The adsorption kinetic of CV onto the PPAC/TG hydrogel biocomposite was well described by the pseudo-second-order model with R^2^ values more than 0.999 and r-χ^2^ values less than 12. Thermodynamic studies confirmed that the CV dye adsorption was spontaneous and endothermic. Furthermore, the prepared hydrogel exhibited excellent reusability, retaining its adsorption capacity even after being used more than five times. Overall, this study concludes that the prepared PPAC/TG exhibited a significant adsorption capacity for cationic dyes, indicating its potential as an effective and eco-friendly adsorbent for water treatment.

## 1. Introduction

Water pollution is a major global problem that endangers both the environment and human health. It occurs when dangerous chemicals enter water bodies, altering aquatic ecosystems and rendering water unfit for consumption [1,2,3]. Among the many contaminants, organic dyes derived from diverse industrial processes have emerged as a concerning contributor to water contamination [4,5]. Textile, paper, and leather industries discharge large volumes of organic dyes into water sources, altering water quality and creating a significant problem for water treatment facilities [6,7]. The leakage of water contaminated with organic dyes not only affects the aesthetic appearance of water bodies but also has harmful effects on aquatic life and human well-being. Addressing the problem of water pollution caused by organic dyes requires sustainable practices and innovative treatment technologies to protect water resources as well as the reuse of treated water in agricultural and other activities [8]. Recent advances in water treatment research have yielded a variety of effective techniques for removing organic dyes, but these techniques vary in terms of cost, efficiency, and the potential to generate secondary pollutants [9,10]. Because of its efficiency and versatility, the adsorption technique has attracted a lot of attention among other approaches [11]. Adsorption is the attachment of dye molecules to the surface of solid materials, which effectively reduces their concentration in water. This procedure has various advantages, including ease of use, low cost, and the ability to remove a wide spectrum of dyes [12]. Many materials have been studied as adsorbents, with biomass, activated carbon, and hydrogels being the most commonly used due to their unique properties [13,14,15,16,17,18].

Hydrogels have emerged as particularly promising adsorbents in recent years [19]. Hydrogels are 3D cross-linked polymer networks that can absorb and hold a lot of water. Adsorbent-based hydrogels are appealing for the removal of pollutants from contaminated water because of their high water retention capacity, tunable characteristics, and reusability [20]. The development of effective bioadsorbents, such as hydrogels, is critical to tackling the problem of organic dye pollution, supporting sustainable water treatment practices and protecting water resources. While hydrogels have shown potential as organic dye adsorbents, they do present some problems that must be addressed. One major difficulty is that their mechanical characteristics are weak, which limits their practical application as adsorbents [21]. Under mechanical stress, hydrogels are frequently brittle and prone to distortion or breakdown, reducing their efficiency and durability. Furthermore, hydrogels exhibit considerable swelling due to the absorption of a large amount of water, which might make recycling polluted water problematic. The extreme swelling of hydrogels demands extra processes to recover the adsorbed dyes, such as squeezing or centrifugation, which use more energy and raise the overall cost of the treatment process [22]. These constraints emphasize the need for additional research and development to improve the mechanical strength and stability of hydrogels as adsorbents, allowing them to be used in practical and efficient water treatment applications [23]. To tackle the difficulties involved with using hydrogels as adsorbents, researchers have investigated several strategies for enhancing their characteristics and controlling their swelling behavior while preserving or improving their adsorption efficiency. Incorporating additional components into the hydrogel framework is one option. One such approach is to incorporate other materials into the hydrogel network to form hydrogel composites. For example, the incorporation of clay, carbon nanomaterials, and metal oxide nanoparticles into hydrogels can improve their mechanical strength and stability, addressing their poor mechanical qualities [24].

Hydrogel composites have gained great interest in recent years, especially those hydrogels based on biopolymers. Biopolymers produced from natural sources have various advantages, including biocompatibility, biodegradability, and sustainability [25]. Tragacanth gum (TG) is a well-known biopolymer utilized in hydrogel biocomposites [26]. TG, which is extracted from the sap of several Astragalus plant species, has unique qualities that make it suited for hydrogel applications [27]. It, like other polar multifunctional gel materials, has a high water absorption capacity, which might result in water retention when utilized as a contaminated water adsorbent. Furthermore, its excessive swelling and poor mechanical qualities make it difficult to remove and reuse after adsorption. Etemadinia et al. examined the removal of Congo red dye from aqueous solutions using a unique adjustable hydrogel composite based on ZnFe_2_O_4_/SiO_2_/TG [28]. The composites were shown to be highly successful in removing Congo red dye, with an adsorption capacity of 159.90 mg/g. The hydrogel composite may also be easily regenerated and reused up to five times without losing substantial adsorption capability. Marziyeh et al. developed a TiO_2_-TG hydrogel composite with a low cost and good adsorption capacity for the removal of methylene blue [29]. The adsorption capacity of the hydrogel composite was 83.82 mg/g, but it was slightly more than the adsorption capacity of TG without TiO_2_, 78.24 mg/g. Sahraei et al. developed a magnetic adsorbent hydrogel using modified TG, polyvinyl alcohol, and graphene oxide [30]. The prepared hydrogel composite exhibited good adsorption capacities, with a maximum adsorption of 94.0 mg/g for crystal violet dye and 101.74 mg/g for Congo red dye. To the best of our knowledge, there is no published study on the enhancement of the adsorption capacity of hydrogel based on TG with activated carbon derived from pomegranate peels for dye removal. Pomegranate-peel-based activated carbon (PPAC) can be regarded as a more cost-effective alternative to traditional activated carbon materials. It provides a renewable and abundant source for the manufacturing of adsorbents, minimizing dependency on fossil-fuel-based precursors. Furthermore, it has the ability to add value to agricultural byproducts, fostering a circular economy approach.

In this study, we have successfully prepared a hydrogel biocomposite based on tragacanth gum (TG) and low-cost activated carbon derived from pomegranate peel waste (PPAC) by a simple method. The results showed that the incorporation of activated carbon (PPAC) contributes to the formation of the hydrogel biocomposite (PPAC/TG) and improves its stability in water as well as its adsorption capacity in crystal violet dye removal from polluted water. Furthermore, various factors influencing the adsorption process were investigated as well as conducting isothermal and kinetic studies using different nonlinear models. Additionally, the thermodynamics of crystal violet dye adsorption on the surface of the PPAC/TG biocomposite and its reusability over six consecutive cycles were evaluated.

## 2. Results and Discussion

### 2.1. Characterization

**Figure 1:**
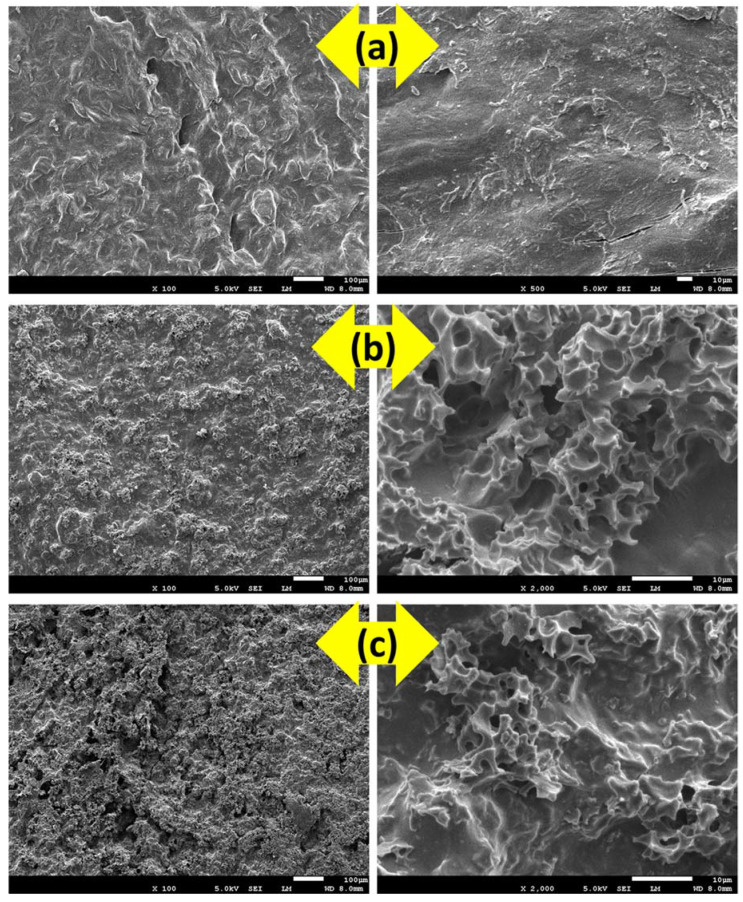
SEM images of (**a**) TG, (**b**) the PPAC/TG hydrogel biocomposite, and (**c**) the PPAC/TG hydrogel biocomposite after adsorption of CV dye.

Figure 1 shows SEM images of TG before and after the incorporation of PPAC (PPAC/TG) as well as after CV dye adsorption onto PPAC/TG. The SEM image (Figure 1a) for pure TG displayed irregular wrinkles, a relatively smooth surface with minimal roughness, and an absence of noticeable pores. In contrast, the morphology of TG after the incorporation of PPAC displays a distinct transformation, as it becomes adorned with highly porous PPAC particles (Figure 1b). These particles introduce a rough texture and contribute to the development of a porous structure on the surface of the PPAC/TG hydrogel that significantly enhances its adsorption performance. After CV dye adsorption onto PPAC/TG (Figure 1c), its surface becomes less porous and rough compared to before adsorption. This observation suggests that the CV dye molecules have been successfully adsorbed onto the surface of PPAC/TG, filling the available pore spaces. As a result, the surface appears smoother, indicating the effective adsorption of the dye molecules.

**Figure 2:**
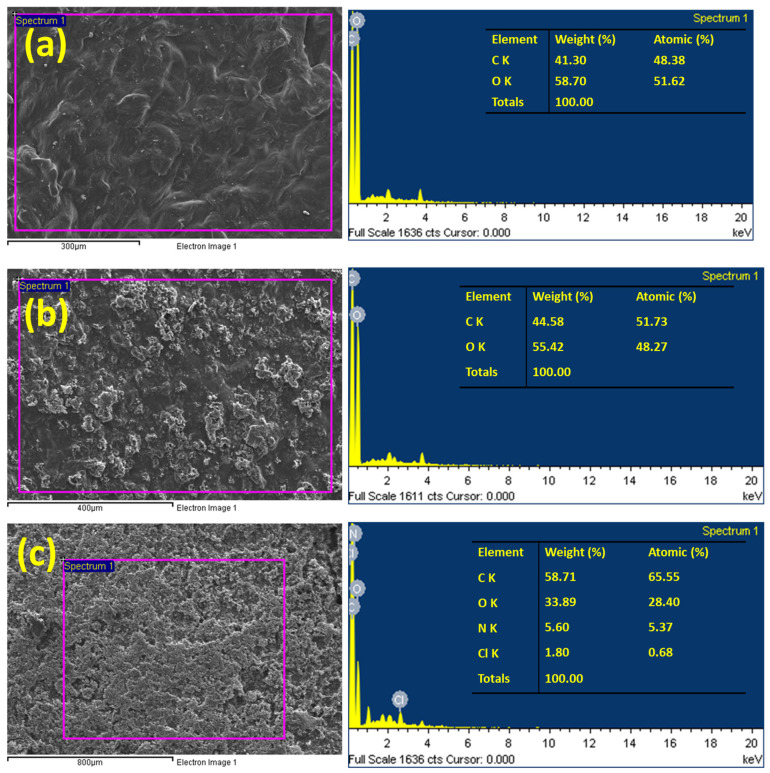
EDS analysis of (**a**) TG, (**b**) the PPAC/TG hydrogel biocomposite, and (**c**) the PPAC/TG hydrogel biocomposite after adsorption of CV dye.

Figure 2 exhibits the EDS analysis, providing valuable insights into the elemental composition of TG, PPAC/TG, and PPAC/TG after adsorption of CV dye. The EDS reveals that TG primarily consists of two elements: carbon and oxygen, with a weight ratio of 41.30% and 58.70%, respectively. The elemental composition changes noticeably after PPAC is incorporated. The carbon percentage rises from 41.3% to 44.58%, suggesting that PPAC has been successfully incorporated into TG. This rise in carbon content demonstrates that carbon particles have been effectively incorporated onto the network of TG. After the dye was adsorbed on the surface of PPAC/TG, it was observed that new elements such as nitrogen and chlorine appeared at a ratio of 5.6 to 1.8%, as well as an increase in the percentage of carbon to 58.71%. This confirms the successful adsorption of the dye and the consequent change in the surface morphology of PPAC/TG.

**Figure 3:**
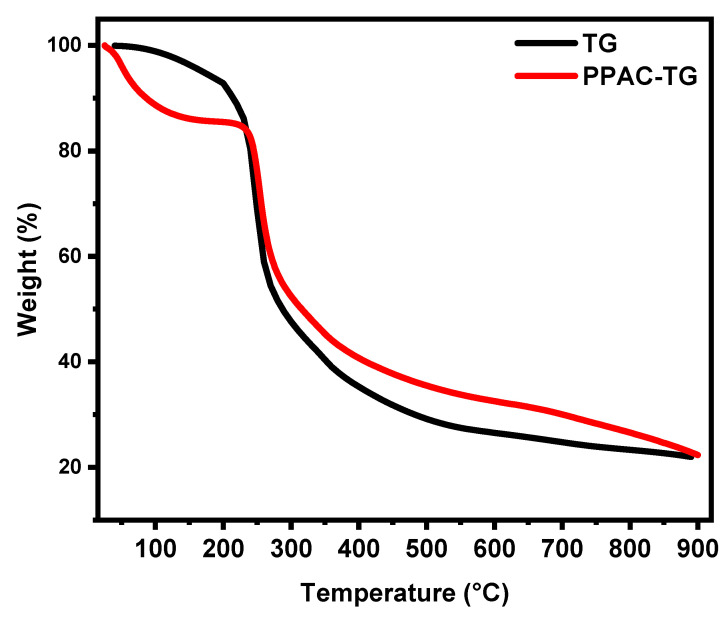
TGA analysis of TG and the PPAC/TG hydrogel biocomposite.

Figure 3 shows the TGA analysis of pure TG and after the incorporation of PPAC (PPAC/TG). The TGA graph shows that pure TG loses weight in three stages. The first stage occurs between 30 and 190 °C and is due to the loss of moisture. The percentage of weight loss in this stage does not exceed 6.5%. The second stage of weight loss occurs between 190 and 330 °C and is due to the decomposition of TG. The weight loss in this stage was 50.5%. The third stage occurs after 350 °C, and the residual weight at 600 °C was 26.5%. The TGA graph of PPAC/TG shows a different behavior degradation compared to pure TG. The weight lost in the first step, which occurs between 30 and 70 °C, reaches 14%. This suggests that PPAC/TG contains a higher moisture content. In the second step, between 170 and 360 °C, the weight loss is 41%, which is attributed to the breakdown of TG and the decomposition of the oxygenated functional groups on the PPAC surface. The thermal stability of PPAC/TG during the second step is higher than that of TG. The third step takes place after 350 °C, and the remaining weight at 600 °C is 32%.

To evaluate the enhanced stability of the thermally treated PPAC/TG composite, four film samples were studied: pure TG, unannealed TG, unannealed PPAC/TG, and annealed PPAC/TG at 150 °C. A total of 20 mg of film sample was immersed in 10 mL of distilled water and shaken for 24 h at 25 °C, and the remaining weight percentage of each sample was measured as an indicator of its stability. After 24 h, pure TG, unannealed TG, and unannealed PPAC/TG were completely degraded. In contrast, annealed PPAC/TG was swelling by 12.5 g/g while keeping the film shape, and the loss weight was approximately 5%. The improvement in the stability of annealed PPAC/TG can be attributed to the thermal cross-link between TG and PPAC.

### 2.2. Adsorption Study

**Figure 4:**
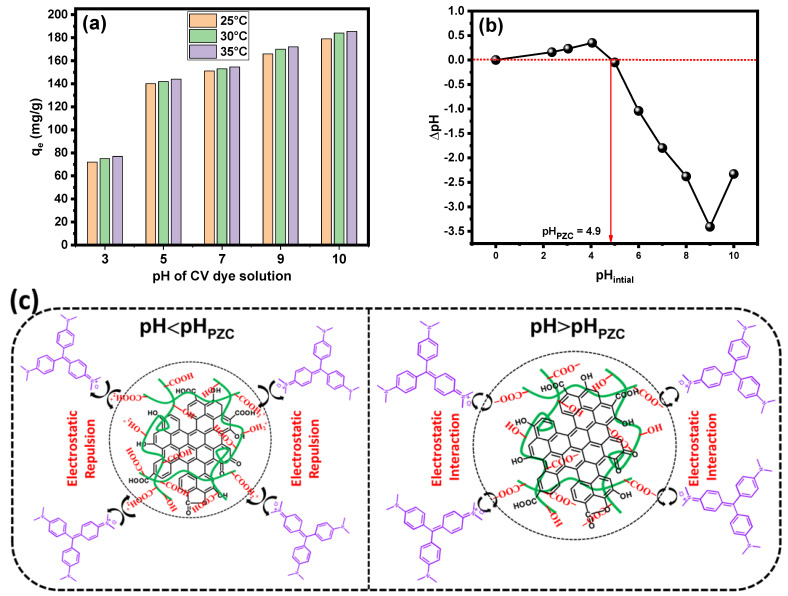
(**a**) pH effect on the adsorption of CV dye onto the PPAC/TG hydrogel biocomposite surface (C_o_ = 200 mg/L, dose = 1 g/L, time = 24 h) at 25, 30, and 35 °C: (**b**) point of zero charge (pH_PZC_) value for the PPAC/TG hydrogel biocomposite and (**c**) the electrostatic repulsion and interaction between CV dye and the PPAC/TG hydrogel biocomposite at a pH < pH_PZC_ and a pH > pH_PZC_, respectively.

The adsorption process is extremely sensitive to changes in the pH of the solution. The type of the ions present in the reaction mixture and their electrostatic interactions with the adsorption surface are principally responsible for this heterogeneity [31]. Furthermore, pH affects the degree of ionization of dissolved species, the adsorbent’s surface charge, and the dissociation of functional groups on the adsorbent’s active sites. The effect of pH on the removal of CV dye by the PPAC/TG hydrogel biocomposite was investigated to acquire a better understanding of the adsorption process. Figure 4a shows the effect of pH and temperature on the PPAC/TG hydrogel biocomposite adsorption capacity. It is noted that the adsorption capacity of the PPAC/TG hydrogel biocomposite increases with increasing pH, and the highest value of adsorption capacity is reached at a pH of 10. On the other hand, it was observed that the adsorption capacity increases with increasing temperature from 25 to 35 °C at various pH values. The point of zero charge (pH_PZC_) value for the PPAC/TG hydrogel biocomposite was determined to be 4.9, as shown in Figure 4b. This pH_PZC_ value indicates that the PPAC/TG hydrogel at a pH value below its respective pH_PZC_ possesses a positive surface charge, while at a pH value above its pH_PZC_, it exhibits a negative surface charge. At lower pH values, more protons are available that can compete with the ionized CV dye molecules for adsorption on the PPAC/TG hydrogel biocomposite surface. In addition, below a pH of 4.9 (pH < pH_PZC_), the PPAC/TG hydrogel biocomposite surface is positively charged, and thus there appears to be an electrostatic repulsion of the positively charged cationic dye molecules from the hydrogel biocomposite surface, as shown in Figure 4c. Above a pH of 5 (pH > pH_PZC_), the surface of the PPAC/TG hydrogel biocomposite becomes more negative due to functional groups (–COOH and –OH) being converted to –COO^–^ and –OH^–^, resulting in more negative sites on the external surface, and a complex may form by the interaction of one CV dye cation and one of these negative sites of the PPAC/TG hydrogel biocomposite. As a result, at higher pH, electrostatic interactions between CV dye molecules and the PPAC/TG hydrogel biocomposite predominate.

**Figure 5:**
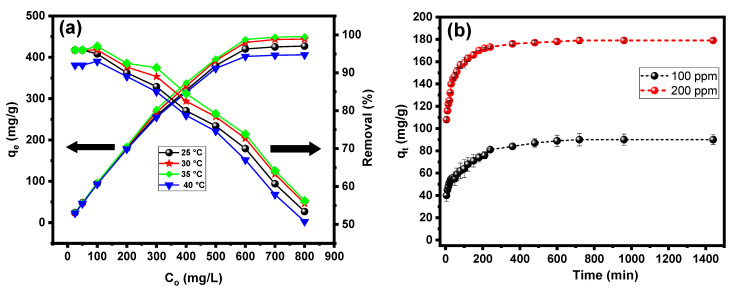
(**a**) Initial concentration effect on removal efficiency and adsorption capacity and (**b**) contact time effect on the adsorption capacity of the PPAC/TG hydrogel biocomposite.

The effect of CV dye concentration on the adsorption capacity of the PPAC/TG hydrogel biocomposite was examined, as displayed in Figure 5a. At first, the adsorption capacity increased proportionally with increasing CV dye concentration in the range of 25 to 600 mg/L at different temperatures. However, after a specific concentration threshold was achieved, the adsorption capacity plateaued. This is due to the most of adsorption sites on the surface of the PPAC/TG hydrogel biocomposite being occupied, which becomes saturated as the dye concentration approaches a specific threshold. It was also noted that the adsorption capacity increased with increasing temperature from 25 to 35 °C. This is due to the dye molecules’ increased kinetic energy as well as the decreased viscosity of the dye solution, which facilitates contact with the adsorption sites. However, at 40 °C, the adsorption capacity began to decline. This decrease can be due to the release of molecules of CV dye from the surface and probable structural changes within the adsorbent material at higher temperatures, which result in a decrease in adsorption effectiveness. Furthermore, the PPAC/TG hydrogel biocomposite demonstrated an impressive ability to completely remove CV dye, as evidenced by the high dye removal efficiency of 96% at concentrations between 25 and 100 mg/L. However, as the dye concentration increased to 800 mg/L, the clearance rate dropped to 50%. This decrease in removal efficiency can be due to the saturation of accessible adsorption sites and greater competition for binding sites among increasing concentrations of dye molecules, resulting in a lower removal efficiency.

The influence of contact duration on the adsorption process, as shown in Figure 5b, was studied at two different dye concentrations. At 100 mg/L, the adsorption capacity increased rapidly with an increasing contact duration, ranging from 5 to 40 min. The removal efficiency reached 56% during the 40 min. Following that, the rate of increase in adsorption capacity was more moderate, reaching equilibrium after 480 min. At a concentration of 200 mg/L, similar behavior was seen, albeit with a higher efficiency of rise in the first phase. During the 40 min contact time, the dye removal effectiveness was 70%. Following this first interval, the adsorption capacity increased until it reached equilibrium after around 360 min. The availability of adsorption sites on the surface of the PPAC/TG hydrogel biocomposite can explain the observed behavior. A large number of empty sites are readily available for CV dye molecules to bind during the early stages of contact time, resulting in a rapid rise in adsorption capacity. As contact time passes, more dye molecules are adsorbed onto accessible sites, resulting in a slower rate of growth in adsorption capacity. When the rate of adsorption equals the rate of desorption, equilibrium is established, resulting in a steady adsorption capacity. The higher initial rate of rise observed at higher dye concentrations can be attributed to a greater number of dye molecules available for adsorption, resulting in the faster saturation of available sites and higher removal rates during the early phases of contact time.

### 2.3. Isotherm Analysis

**Figure 6:**
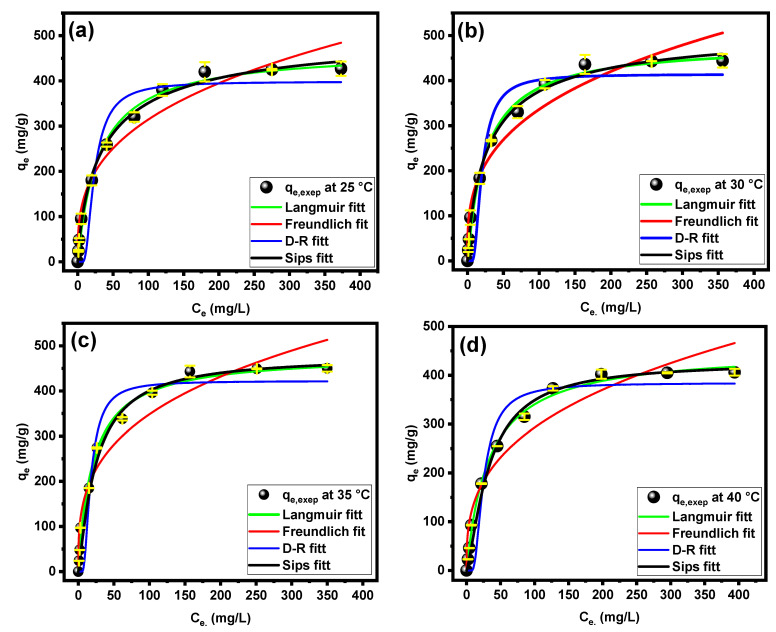
Nonlinear isotherm models for CV dye onto the PPAC/TG hydrogel biocomposite at (**a**) 25 °C, (**b**) 30 °C, (**c**) 35 °C, and (**d**) 40 °C.

For modeling the experimental data of equilibrium adsorption for CV dye onto the PPAC/TG hydrogel biocomposite at various temperatures (25, 30, 35, and 40 °C), the adsorption isotherms of CV dye were examined. To fit the experimental data, three nonlinear isotherm models (Langmuir, Freundlich, Dubinin–Radushkevich, and Sips) were used, as shown in Figure 6a–c. The details of the equations for the used isotherm models can be found in the Supplementary Materials (Equations (S6)–(S11)). Table 1 shows the related isotherm parameters. To assess the goodness of fit of each model, a number of statistical error functions were used. As shown in Table 1, the Langmuir model and Sips matched the experimental data quite well, as shown by the very high R^2^ values (more than 0.99). However, the Sips model offered the best fit among the several dye adsorption models for the PPAC/TG hydrogel biocomposite due to its superior correlation: all χ^2^ values were determined to be less than 64. Moreover, the maximum adsorption capacity values according to the Sips isotherm model were closer to the experimental adsorption capacity values. The Sips isotherm is a combination of the Langmuir and Freundlich isotherm models that is intended to characterize heterogeneous surfaces better. It behaves like a Freundlich isotherm at low adsorbate concentrations but predicts a monolayer adsorption capacity comparable to the Langmuir isotherm at high adsorbate concentrations. According to the Sips model, the maximum adsorption capacity (q_max_) reached 455.61, 470.86, 477.31, and 427.53 mg/g at temperatures of 25, 30, 35, and 40 °C, respectively. An analysis of the constant Sips (K_s_) values presented in Table 1 revealed a significantly higher affinity for the CV dye to the adsorbent with increasing temperature. The heterogeneity factor (n_s_) values also exceeded unity, indicating that the adsorption processes were heterogeneous in nature. The same behavior was found in the study conducted on the adsorption of MB dye on modified β-cyclodextrin, where the *n* values were higher than unity [32].

Table 2 compares the q_max_ of the PPAC/TG hydrogel biocomposite for CV dye with other hydrogel-based adsorbents. According to the q_max_ results, the PPAC/TG hydrogel biocomposite had a high adsorption capacity when compared to other reported adsorbents [33,34,35,36,37,38,39,40]. These findings suggest that the PPAC/TG hydrogel biocomposite is a viable adsorbent for removing cationic dyes.

### 2.4. Kinetic Analysis

**Figure 7:**
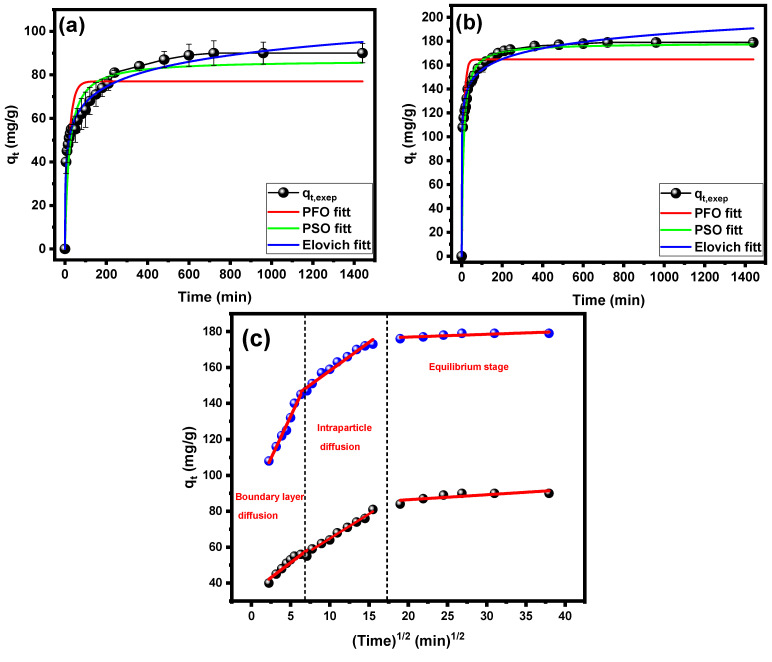
Nonlinear kinetic models for CV dye onto the PPAC/TG hydrogel biocomposite at concentrations of (**a**) 100 mg/L and (**b**) 200 mg/L, and (**c**) the intraparticle diffusion model.

To evaluate the kinetics of CV dye adsorption onto the PPAC/TG hydrogel biocomposite, the pseudo-first-order (PFO), pseudo-second-order (PSO), Elovich, and intraparticle diffusion models were employed to fit the experimental data. The details of the equations for the used kinetic models can be found in the Supplementary Materials (Equations (S12)–(S15)). Figure 7a,b illustrates the nonlinear curves of the PFO, PSO, and Elovich kinetic models for the adsorption of CV dye with a concentration of 100 and 200 mg/L, respectively. The parameter estimations and determination R^2^ and χ^2^ are summarized in Table 3. The equilibrium adsorption capacity (q_e_) of 100 and 200 mg/L CV dye was determined experimentally to be 90 and 179 mg/g, respectively. The PSO model estimated adsorption capacity values at 100 and 200 mg/L to be 86.73 and 178.42 mg/g with errors of 3.63 and 0.32%, while the error in adsorption capacity value estimated by the PFO was 14 and 8%, respectively. Furthermore, as compared to the PFO and Elovich models, the PSO model showed much higher R^2^ values and lower χ^2^ values. This shows that the PSO model fits the experimental data better and is more accurate in describing the adsorption process. Similar adsorption behavior for PPAC/TG hydrogel composites have been reported for other systems, including GO/xanthan gum hydrogel [41], montmorillonite/poly(acrylamide-co-maleic acid) composite [42], and hydrogel based on polyacrylamide/sodium alginate/2-acrylamido-2-methylpropane sulphonic acid [43].

The intraparticle diffusion model is commonly used to estimate the rate-controlling step, which is mostly influenced by surface or pore diffusion. The plot of q_t_ versus t^0.5^ for the intraparticle diffusion model in Figure 7c aids the understanding of the number of phases in the adsorption process. The CV dye adsorption process can be separated into three distinct stages, as shown in Figure 7c. The initial stage shows a significant increase in adsorption, which can be attributable to the rapid attachment of CV molecules to the exterior surface of the PPAC/TG hydrogel biocomposite. The diffusion of CV molecules within the adsorbent intraparticle is the rate-limiting process in the second stage of adsorption. Finally, the third stage marks the point at which CV molecules have almost entirely covered the active sites on the PPAC/TG hydrogel biocomposite surface. Due to the extremely low residual concentrations of CV dye in the solution, intraparticle diffusion slows dramatically during this stage [44,45]. The plot of qt vs. t^0.5^ should show a linear connection that does not pass through the origin to establish that intraparticle diffusion does not dominate CV adsorption onto the PPAC/TG hydrogel biocomposite. This conclusion is compatible with previous research, such as the adsorption of CV onto hydrogel-based adsorbents [46,47].

### 2.5. Thermodynamic Study

Thermodynamic studies provide useful information on the effect of temperature changes on the adsorption process. To understand the nature and spontaneity of the sorption process for CV dye onto the PPAC/TG hydrogel biocomposite, various thermodynamic parameters, including enthalpy (∆H^o^), entropy (∆S^o^), and Gibbs free energy (∆G^o^), were calculated. The mathematical formulae for these parameters are given in the following Equations (1)–(3).

(1) ΔG°=−RTlnKeq

(2) $${\mathrm{lnK}}_{c}=\frac{\Delta S{}^{\circ}}{R}-\frac{\Delta H{}^{\circ}}{\mathrm{RT}}$$

(3) $$K_{c}=K_{s}\times \;M.W\;\times 55.5\times 1000$$

K_c_ is a constant in the equations that expresses the ratio of the amount of dye adsorbed on the adsorbent surface to the equilibrium dye concentration and is calculated by using the Sips model constant (K_s_). T is the Kelvin (K) temperature, and R is the universal gas constant (8.314 J/mol K). The slope and intercept of the lnK_c_ against 1/T plot, also known as the van’t Hoff plot, were used to calculate ∆H^o^ and ∆S^o^. The results of these calculations are summarized in Table 4. The results in Table 4 show that the adsorption of CV onto the PPAC/TG hydrogel biocomposite is characterized by a positive value of ∆H^o^, indicating that the process is endothermic. Negative values of Gibbs free energy (∆G^o^) imply that the adsorption processes for CV dye are feasible and spontaneous [48]. The positive ∆S^o^ value indicates the PPAC/TG hydrogel biocomposite has a significant affinity for CV dye, as well as an increased unpredictability at the solid–solution interface during the sorption process.

### 2.6. Adsorption Mechanism and Reusability

**Figure 8:**
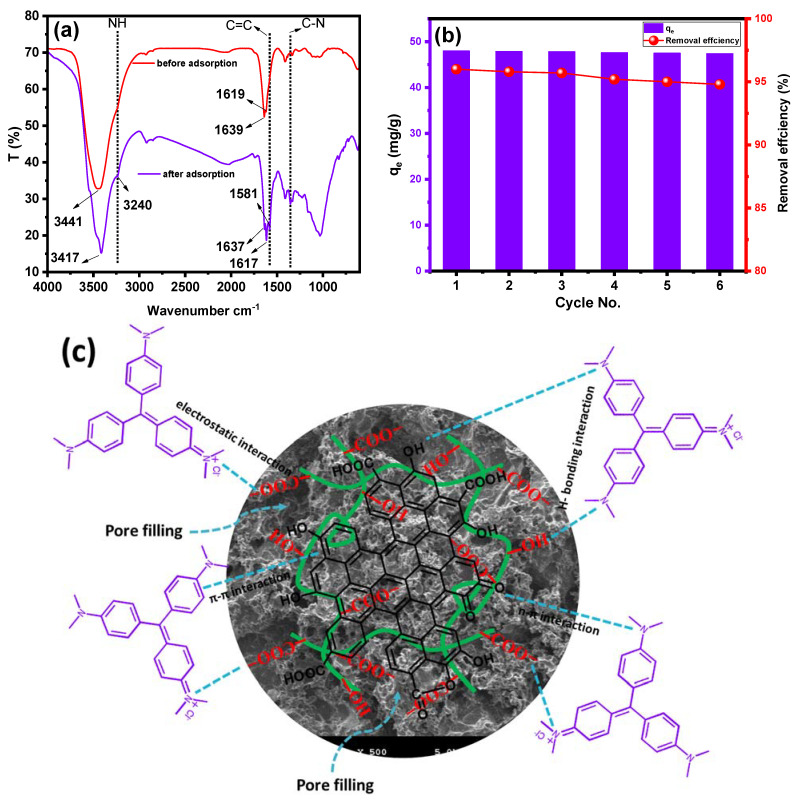
(**a**) FTIR of the PPAC/TG hydrogel biocomposite before and after CV dye adsorption and (**b**) the reusability of PPAC/TG and (**c**) proposed adsorption mechanism.

The FTIR spectrum (Figure 8a) of the PPAC/TG hydrogel biocomposite exhibits absorption bands at 3441, 1639, and 1386 cm^−1^, corresponding to the O-H stretching, asymmetrical, and symmetrical stretching vibrations of the –COO^−^ group, respectively [40,49]. Additionally, a weak absorption band at 1619 cm^−1^ corresponds to the stretching vibration of the –COOH groups in PPAC. After the adsorption of CV dye, there is noticeable changes in the absorption wavenumbers and intensities of the PPAC/TG hydrogel biocomposite as well as new bands. Slight shifts in peak positions were observed for bands of -OH and -COO^−^ groups, indicating the removal of CV over the PPAC/TG hydrogel biocomposite through hydrogen bonds and electrostatic interactions between the N-H groups of the CV dye molecules and the –OH and –COO^−^ groups of the PPAC/TG hydrogel biocomposite, respectively. Moreover, the new absorption bands at 1581 and 1355 cm^−1^ are attributed to the C=C of the benzene ring and C-N in CV dye molecules [50]. These findings align with the previously discussed results of the impact of the pH and point zero charge of the PPAC/TG hydrogel biocomposite, which corroborated the role of electrostatic attraction in the adsorption of CV dye. The result demonstrated that adsorption capacity increases with increasing pH and that the surface of the PPAC/TG hydrogel biocomposite exhibits a negative charge at pH values above 4.9. Figure 8c summarizes the most important forces contributing to the adsorption of the CV dye on the PPAC/TG surface, represented by electrostatic attraction, hydrogen bonding, pore filling, π-π interaction, and n-π interaction.

The reuse of the PPAC/TG hydrogel biocomposite as an adsorbent for the removal of CV dye, specifically at a concentration of 50 mg/L, has been explored as displayed in Figure 8b. The results demonstrated that the PPAC/TG hydrogel biocomposite can be reused up to six times without significantly reducing its adsorption capacity. This shows that the adsorption process on the surface of the PPAC/TG hydrogel biocomposite is predominantly governed by physical forces. The capacity of the PPAC/TG hydrogel biocomposite to be reused several times highlights its potential as a cost-effective and environmentally friendly adsorbent. This is particularly significant since the PPAC/TG hydrogel biocomposite is composed of activated carbon, which can be derived from renewable sources such as pomegranate peel waste, and it can also be incorporated into tragacanth gum, further enhancing its sustainability and potential for reusability.

## 3. Conclusions

The synthesis of the PPAC/TG hydrogel biocomposite was successfully achieved using a thermally cross-linked method and then used as an adsorbent for the removal of CV cationic dye. The PPAC/TG hydrogel biocomposite showed a porous structure and high stability in aqueous media with a swelling capacity of 12.55 g/g and a degradation rate of less than 6.0%. The hydrogel biocomposite demonstrates exceptional adsorption performance for CV dye, achieving an adsorption capacity of 477.37 mg/g. Isothermal studies confirmed that the Sips model is the most appropriate to describe the adsorption behavior, compared to the Langmuir and Freundlich model. Temperature plays a significant role in determining the maximum adsorption capacity, initially increasing from 398.48 mg/g to 477.37 mg/g with a rising temperature from 25 °C to 35 °C but then decreasing as the temperature exceeds 40 °C. The pseudo-second-order kinetic model provided the best fit for the adsorption of CV dye onto the PPAC/TG hydrogel biocomposite. The reusability studies demonstrated the remarkable stability of the PPAC/TG hydrogel biocomposite, retaining stable adsorption capacity even after six consecutive cycles. This outstanding adsorption capacity and reusability makes the PPAC/TG hydrogel biocomposite a highly promising material for the remediation of organic cationic dyes from contaminated water.

## 4. Materials and Method

### 4.1. Materials

Tragacanth gum and pomegranate peels were obtained from the local market of the city of Riyadh and sourced from India and Yemen, respectively. Crystal violet dye (C_25_H_30_ClN_3_) (ACS, Reag. Ph Eur, ≥90%), sodium hydroxide (97%), potassium hydroxide (≥85%), and hydrochloric acid (36%) were purchased from Sigma-Aldrich Co, Steinheim Germany.

### 4.2. Preparation Method

Activated carbon was prepared from pomegranate peels in the same method as reported by Thamer et al. [51].

The PPAC/TG hydrogel biocomposite was prepared by the dispersion of 100 mg of PPAC in 50 mL of distilled water in an ultrasonication water bath, and then 1 g of TG was added to the PPAC dispersed solution, followed by stirring for 12 h at 80 °C. The mixture was then poured onto a glass dish and dried in a vacuum oven for three days. Finally, the dried product was heated at 150 °C to promote thermal cross-linking. Except for the exclusion of PPAC in the preparation process, an identical method was followed for preparing the tragacanth hydrogel. The same method was followed to prepare the tragacanth gum hydrogel, except for the addition of PPAC.

### 4.3. Adsorption Measurements

Due to the effect of pH on both the adsorbent and adsorbate, a study was initiated to determine the optimal pH to achieve the highest adsorption capacity of the CV dye onto the PPAC/TG hydrogel surface. To investigate the effect of pH, CV solutions with pH values of 3, 5, 7, and 10 were prepared. A fixed amount of 10 mg of PPAC/TG hydrogel was added to 10 mL of each dye solution, and the mixtures were then placed in a shaker set at 25 °C and 120 rpm for 24 h to ensure equilibrium. After 24 h, the PPAC/TG hydrogel was picked up with tweezers, and the residual concentration of the CV dye in the solution was determined using a UV–VIS spectrophotometer (Perkin-Elmer-Lambda35, Caerphilly, UK) at 589 nm. To conduct adsorption equilibrium studies, 10 mg of PPAC/TG was added to 10 mL of CV dye solution with an initial concentration range of 25 to 800 mg/L and a pH of 10. The study of the effect of concentration was repeated under the same conditions and at temperatures of 30, 35, and 40 °C. Adsorption studies also were carried out with two different CV dye concentrations (100 ppm and 200 ppm) at a constant pH of 10 and 25 °C to study the influence of contact time. The residual concentration of CV dye was measured at various time intervals ranging from 5 min to 24 h. In order to conduct a reusability investigation, 10 mg of PPAC/TG was mixed into a 10 mL solution of CV dye with a concentration of 50 mg/L and a pH of 10. After that, the mixture was stirred for 4.0 h. Following that, the adsorbent was removed from the solution, and the remaining dye concentration was determined. To desorb the CV dye, the adsorbent was mixed with a 10 mL solution of HCl (0.2 M) and acetone in a 75:25 ratio and shaken for 2.0 h. Following that, the PPAC/TG was separated and washed with distilled water before being treated with a dilute solution of NaOH (0.01 M). Finally, the adsorbent was washed with distilled water one more to prepare it for reuse.

The pH drift method was used to estimate the point of zero charge (pH_pzc_) of PPAC/TG. A series of KCl solutions with fixed concentrations of 0.1 M and varying pH values from 2 to 11 was prepared. A total of 20 mg of PPAC/TG was added to each solution, which was then agitated at 120 rpm and 25 °C in a thermostatically controlled water bath shaker. After a 4 h equilibration interval, the final pH solution (pH_f_) was determined using a pH meter (3540, Jenway, UK). The differences between pH_f_ and pH_i_ values (∆pH) were then plotted against the initial pH (pH_i_) values. Calculation of adsorption capacity, Statistical analysis, Adsorption isotherm models and Kinetic Studies of Adsorption are provided in the supplementary material [52,53,54,55,56,57,58,59].

### 4.4. Characterization

A complete characterization of the prepared hydrogel biocomposite was performed by utilizing a variety of techniques. The materials’ surface morphology and microstructure were examined using a scanning electron microscope (SEM, JEOL 2100, Tokyo, Japan), which provided high-resolution imagery and detailed information on their topography. The elemental content and distribution inside the hydrogel biocomposite were determined using energy-dispersive X-ray spectroscopy (EDS) analysis in conjunction with the SEM. Fourier-transform infrared spectroscopy (FTIR, Thermo Scientific, NicoletiS50, Waltham, MA, USA) was used to detect the functional groups contained in the hydrogel biocomposite, allowing chemical bonds and molecular structures to be analyzed. In addition, thermogravimetric analysis (TGA, Q500, New Castle, DE, USA) was used to study the thermal stability and decomposition of the hydrogel biocomposite.

## Figures and Tables

**Table 1:** Adsorption isotherm parameters for CV dye adsorption onto the PPAC/TG hydrogel biocomposite at 25, 30, 35, and 40 °C.

Model	Adsorbent			
	25 °C	30 °C	35 °C	40 °C
q_e,exp_ (mg/g)	427	444	450	406
Langmuir				
q_max_ (mg/g)	468.89 ± 13.25	482.98 ± 12.58	481.21 ± 12.51	452.19 ± 8.25
K_L_ (L/mg)	0.033 ± 0.003	0.0386 ± 0.0042	0.0477 ± 0.0056	0.0304 ± 0.0022
R^2^	0.9923	0.9930	0.9922	0.9967
r-χ^2^	249.68	245.74	285.06	100.11
Freundlich				
KF (mg/g)/(mg/L)^n^	69.51 ± 13.67	76.22 ± 15.25	83.73 ± 16.98	61.59 ± 14.94
1/n	3.05 ± 0.358	3.10 ± 0.3828	3.23 ± 0.4237	2.95 ± 0.4098
R^2^	0.9571	0.9513	0.9437	0.9374
r-χ^2^	1398.01	1720.84	2051.91	1880.34
Dubinin–Radushkevich				
qs (mg/g)	398.48 ± 22.48	414.15 ± 22.70	421.99 ± 21.76	384.06 ± 20.82
K_D-R_ (mol^2^/KJ^2^)	69.36 ± 21.87	49.77 ± 14.90	36.01 ± 9.81	71.59 ± 21.94
E (kJ/mol)				
R^2^	0.9278	0.9321	0.9395	0.9328
r-χ^2^	2352.92	2400.43	2205.74	2017.92
Sips				
q_s_	455.61 ± 16.07	470.86 ± 15.21	477.37 ± 15.05	427.53 ± 10.81
K_s_	0.0122 ± 0.0011	0.0140 ± 0.0099	0.02265 ± 0.0193	0.0089 ± 0.0072
n_s_	1.25 ± 0.2396	1.26 ± 0.2440	1.18 ± 0.2311	1.35 ± 0.2106
R^2^	0.9956	0.9957	0.9993	0.9993
r-χ^2^	63.66	59.76	50.87	44.40

**Table 2:** Comparison of the q_max_ of the PPAC/TG hydrogel biocomposite for CV dye with similar adsorbents.

Adsorbent	Adsorption Conditions	q_max_	Ref.
Kappa-carrageenan-sodium alginate	C_o_ = 10–100 mg/L, pH = 6.4, T = - K	88.8	[33]
GO/polyacrylamide/sodium alginate	C_o_ = 10–300 mg/L, pH = 8, T = 298 K	100.3	[34]
Xanthan gum/poly(N-vinyl imidazole)	C_o_ = 450–600 mg/L, pH = 7, T = 303 K	453	[35]
Poly(acrylic acid-acrylamide-methacrylate)/amylose	C_o_ = 1–50 mg/L, pH = 7.4, T = 298 K	35.09	[36]
Polydopamine/montmorillonite/ pullulan	C_o_ = 50–300 mg/L, pH = -, T = - K	112.45	[37]
Guar gum/bentonite	C_o_ = 5–50 mg/L, pH = 7.6, T = 293 K	167.29	[38]
Karaya gum /montmorillonite	C_o_ = 20–100 mg/L, pH = 7, T = 300 K	137.77	[39]
Tragacanth gum/PVA nanofibers	C_o_ = 25–450 mg/L, pH = 10, T = 298 K		[40]
Activated carbon/tragacanth gum	C_o_ = 25–600 mg/L, pH = 10, T = 298 K	477.37	This study

**Table 3:** Adsorption kinetics parameters for CV dye adsorption onto the PPAC/TG hydrogel biocomposite at 25, 30, 35, and 40 °C.

Model	Concentration	
	100 mg/L	200 mg/L
q_e,exp_ (mg/g)	90	179
Pseudo-first-order		
q_e,cal_ (mg/g)	77.01 ± 3.08	164.81 ± 3.82
k_1_	0.0505 ± 0.0096	0.1050 ± 0.01595
R^2^	0.7333	0.8522
χ^2^	127.05	238.65
Pseudo-second-order		
q_e,cal_ (mg/g)	86.73 ± 2.24	178.42 ± 1.21
K_2_	5.73 × 10^−4^ ± 9.71 × 10^−5^	6.29 × 10^−4^ ± 4.85 × 10^−5^
R^2^	0.9998	0.9999
r-χ^2^	7.65	11.26
Elovich		
β	0.0959 ± 0.0037	0.0769 ± 0.0053
α	66.19 ± 14.51	21,132.93 ± 16,720.17
R^2^	0.9839	0.9998
r-χ^2^	32.67	25.97

**Table 4:** Thermodynamic parameter values for the adsorption of CV dye by the PPAC/TG hydrogel biocomposite.

Temperature (K)	∆G° (KJ/mol)	∆H° (KJ/mol)	∆S° (J/mol K)
298	−31.04	47.06	261.62
303	−31.91		
308	−33.67		

## Data Availability

The data presented in this study are openly available in article.

## Supplementary Materials

The following supporting information can be downloaded at https://www.mdpi.com/article/10.3390/gels9120959/s1, 1. Equations of the calculation of adsorption capacity (Equations (S1)–(S3)), statistical analysis equations (Equations (S4) and (S5)), isotherm model equations (Equations (S6)–(S11)), and kinetic model equations (Equations (S12)–(S15)).
