# Supplementary material for: Activated Carbon-Incorporated Tragacanth Gum Hydrogel Biocomposite: A Promising Adsorbent for Crystal Violet Dye Removal from Aqueous Solutions
Source: Gels. 2023 Dec 7;9(12):959. doi: 10.3390/gels9120959 (PMC10743021; doi:10.3390/gels9120959)
Supplement: Supplementary file 1 [file gels-09-00959-s001.zip › gels-2754233-supplementary.pdf]

Activated carbon-incorporated tragacanth gum hydrogel biocomposite: A promising adsorbent for crystal violet dye removal from aqueous solutions

Badr M. Thamer [1, *], Faiz A. Al-aizari[1] and Hany S. Abdo [2]

[1] Department of Chemistry, College of Science, King Saud University, P.O. Box 2455, Riyadh 11451, Saudi Arabia
[2] Department of Mechanical Engineering, College of Engineering, King Saud University, Riyadh, 11421, Saudi Arabia
* Correspondence: bthamer@ksu.edu.sa

1. Calculation of adsorption capacity

Equations 1-3s were applied to measure the adsorption capacity at specific time t (qt) and equilibrium (q_e) and separation efficiency.

$$q_t = \frac{C_o - C_t}{m} \times V \qquad \text{(S1)}$$

$$q_e = \frac{C_o - C_e}{m} \times V \qquad \text{(S2)}$$

$$Removal\ \ effeciency\ (\%) = \frac{C_o - C_e}{C_o} \times\ 100 \qquad \text{(S3)}$$

C_o (mg/L) is the initial concentration, C_t (mg/L) is the concentration at a specific time, and C_e (mg/L) is the concentration of dye at the equilibrium state. V (L) refers to the used volume of dye solution, and the mass of adsorbents was denoted by *m* (g).

2. Statistical analysis

Recently, nonlinear regression method is one of the most reliable tools to define the best fitting experimental data of adsorption and kinetic process. To determine the best kinetic and isotherm model for representing experimental data, Chi-square values (χ^2) and coefficient of determination values (R^2) were calculated for the nonlinear method by Eq.4

and Eq.6, respectively. The model with the lowest value of χ^2 and the highest value of R^2 is the most representative of the experimental data and the least error.

$$\chi^2 = \sum_i^n \frac{(q_{e,exp} - q_{e,model})^2}{q_{exp}} \qquad \text{(S4)}$$

$$R^2 = 1 - \frac{\sum(q_{e,exp} - q_{e,model})^2}{\sum(q_{e,exp} - q_{e,mean})^2} \qquad \text{(S5)}$$

where ($q_{e,model}$) is each value of q_e predicted by the fitted model, ($q_{e, exp}$) is each value of q_e measured experimentally, ($q_{e,mean}$) is the average value of q_e that was measured experimentally, n_p is the number of performed experiments, and p is the number of parameters for the fitted model.

2. Adsorption isotherm models

3.1. Langmuir isotherm

The Langmuir isotherm model assumes homogeneity of the adsorbent surface and monolayer coverage by the adsorbate. This model can be represented by the following non-linear equation [1]:

$$q_e = \frac{Q_o K_L C_e}{1 + K_L C_e} \qquad \text{(S6)}$$

Where q_e is the amount of adsorbate (mg/g), C_e is the equilibrium concentration of adsorbate in solution (mg L^{-1}), K_L is the Langmuir constant (L mg^{-1}), and Q_o (mg/g) is the monolayer adsorption capacity.

3.2. Freundlich isotherm

The Freundlich isotherm model more accurately describes heterogeneous surfaces or multilayers of adsorbent and can be described as follows [2]:

$$q_e = K_f \, C_e^{1/n} \qquad \text{(S7)}$$

K_f is the Freundlich constant related to the adsorption capacity, and $1/n$ is the constant related to adsorption strength. If the value of $1/n$ is between 0.1 and 1.0, the adsorption is favorable.

3.3. Dubinin–Radushkevich (D-R)

To test the nature of adsorption (physical or chemical) of adsorbate onto the adsorbent surface, the Dubinin-Radushkevich (D-R) isotherm model was applied as follows [3]:

$$q_e = q_o \, e^{-K_{D-R} \, \varepsilon^2} \qquad \text{(S8)}$$

where q_e (mg g^{-1}) is the equilibrium adsorption capacity, q_o (mg g^{-1}) is the maximum adsorption capacity, $K_{D\text{-}R}$ (mol^2 kJ^{-2}) is the activity coefficient related to the mean free energy of adsorption, and ε is the Polanyi potential, which can be calculated from the following equation:

$$\varepsilon = RTln(1 + \frac{1}{C_e}) \qquad \text{(S9)}$$

where R (J mol^{-1} K^{-1}) is the gas constant, and T (K) is the absolute temperature. For the D-R isotherm model, from the $K_{D\text{-}R}$ values, the mean energy, E, can be calculated using the following equation:

$$E = \frac{1}{\sqrt{2K_{D-R}}} \qquad \text{(S10)}$$

If E is between 8 and 16 kJ mol^{-1}, adsorption is achieved by chemical processes, whereas when $E < 8$ kJ mol^{-1} physical processes dominate.

3.4. Sips model

The Sips isotherm model, developed in 1948, was designed to address the issue of continuous increase in the adsorbed amount with increasing concentration, a phenomenon commonly observed in the Freundlich isotherm model. The Sips isotherm model is mathematically represented by the following equation [4]:

$$q_e = \frac{q_s K_s C_e^n}{1+K_s C_e^n} \qquad \text{(S11)}$$

where q_s is the Sips maximum adsorption capacity (mg/g), K_s is the Sips constant $(L/mg)^n$, and n is the Sips model exponent.

3. Kinetic Studies of Adsorption

The adsorption rate is another important factor to optimum operating condition as it provides valuable information about the reaction pathways and the mechanism of adsorption reactions. There are many kinetic models which have been developed for the determination of adsorption rate. The most commonly used are Pseudo-first-order model (PFO), Pseudo-second-order model (PSO), Elovich model and intra-particle diffusion model.

5.1 Pseudo-first-order model (PFO)

PFO model is a nonlinear relationship and it can be represented as follow [5]:

$$q_t = q_e(1 - e^{-K_1 t}) \qquad \text{(S12)}$$

In which, q_t and q_e are the amounts of adsorbate adsorbed at time t and equilibrium, respectively, K_1 (min^{-1}) is the rate constant of the PFO.

5.2 Pseudo-second-order model (PSO)

PSO can be inferred from this model that the chemisorption is vital in the rate determining step (Ho and McKay, 1999). The nonlinear form of pseudo-second-order model can be expressed as [6]:

$$q_t = \frac{q_2^2 k_2 t}{1 + q_e K_2 \ t} \qquad \text{(S13)}$$

Where K_2 (g mg^{-1} min^{-1}) is the rate constant of the PSO adsorption process.

5.3 Elovich kinetic model

The Elovich kinetic model is another model that can be applied for the study of the chemisorption. In the absence of desorption, the reaction rate will decrease owing to the increasing surface coverage. The equation can be shown as [7]:

$$q_t = \frac{1}{\beta}\ln(1 + \alpha\beta t) \qquad \text{(S14)}$$

Where α (mg/g min^{-1}) is the initial adsorption rate; β (mg/g) is desorption constant during any one experiment.

5.4 Intra-particle diffusion

To determine the steps involved in the adsorption process, the Weber-Morris intraparticle diffusion model was used to further analyze the kinetic results. This model is described by the following equation [8]:

$$q_t = K_p\, t^{0.5} + C \qquad \text{(S15)}$$

where k_p (mg/g min$^{-1/2}$) and C (mg g^{-1}) represent the intraparticle diffusion rate constant and the constant related to the thickness of the boundary layer, respectively, which can be determined from the slope and intercept of the q_t versus $t^{0.5}$ plot.

References:

1. Langmuir, I. The adsorption of gases on plane surfaces of glass, mica and platinum. *J. Am. Chem. Soc.* **1918**, *40*, 1361–1403, doi:10.1021/ja02242a004.
2. Freundlich, H.M.F. Over the adsorption in solution. *Z. Phys. Chem* **1906**, *57*, 385–471.
3. Dubinin, M., Radushkevich, L. Equation of the characteristic curve of activated charcoal. *Chem. Zentr* **1947**, *1*, 875–890.
4. Foo, K.Y.; Hameed, B.H. Insights into the modeling of adsorption isotherm systems. *Chem. Eng. J.* **2010**, *156*, 2–10, doi:10.1016/J.CEJ.2009.09.013.
5. Lagergren, S. About the theory of so-called adsorption of soluble substances. *K. Sven. Vetenskapsakademiens Handl.* **1898**, *24*, 1–39.
6. Ho, Y.S.; Wase, D.A.J.; Forster, C.F. Kinetic Studies of Competitive Heavy Metal Adsorption by Sphagnum Moss Peat. *Environ. Technol.* **1996**, *17*, 71–77, doi:10.1080/09593331708616362.
7. Chien, S.H.; Clayton, W.R. Application of Elovich Equation to the Kinetics of Phosphate Release and Sorption in Soils1. *Soil Sci. Soc. Am. J.* **1980**, *44*, 265, doi:10.2136/sssaj1980.03615995004400020013x.

8. WJ Weber, J.M. Kinetics of Adsorption on Carbon from Solution. *J. Sanit. Eng. Div.* **1963**, *89*, 31–60.
